# Supplementary material for: Recording site placement on planar silicon-based probes affects signal quality in acute neuronal recordings
Source: Sci Rep. 2021 Jan 21;11:2028. doi: 10.1038/s41598-021-81127-5 (PMC7819990; doi:10.1038/s41598-021-81127-5)
Supplement: Supplementary file 1 — Supplementary Information. [file 41598_2021_81127_MOESM1_ESM.pdf]

Supplementary Material

Recording site placement on planar silicon-based probes affects signal quality in acute neuronal recordings

Richárd Fiáth[1,2*], Domokos Meszéna[1,2], Zoltán Somogyvári[3], Mihály Boda[2], Péter Barthó[1], Patrick Ruther[4,5], István Ulbert[1,2]

[1] Institute of Cognitive Neuroscience and Psychology, Research Centre for Natural Sciences, Budapest, Hungary

[2] Faculty of Information Technology and Bionics, Pázmány Péter Catholic University, Budapest, Hungary

[3] Department of Computational Sciences, Wigner Research Centre for Physics, Budapest, Hungary

[4] Department of Microsystems Engineering (IMTEK), University of Freiburg, Freiburg, Germany

[5] Cluster of Excellence, BrainLinks-BrainTools, University of Freiburg, Freiburg, Germany

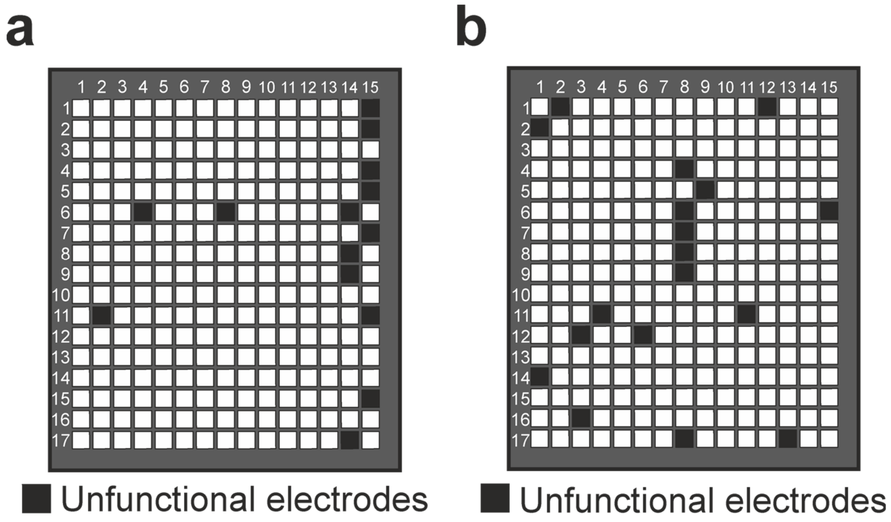

Supplementary Figure 1. The position of unfunctional recording sites/channels (black squares) on the 255-channel silicon probe used in the study (a), and on the 255-channel probe which was used to collect the online available data ((b); www.kampff-lab.org/ultra-dense-survey).

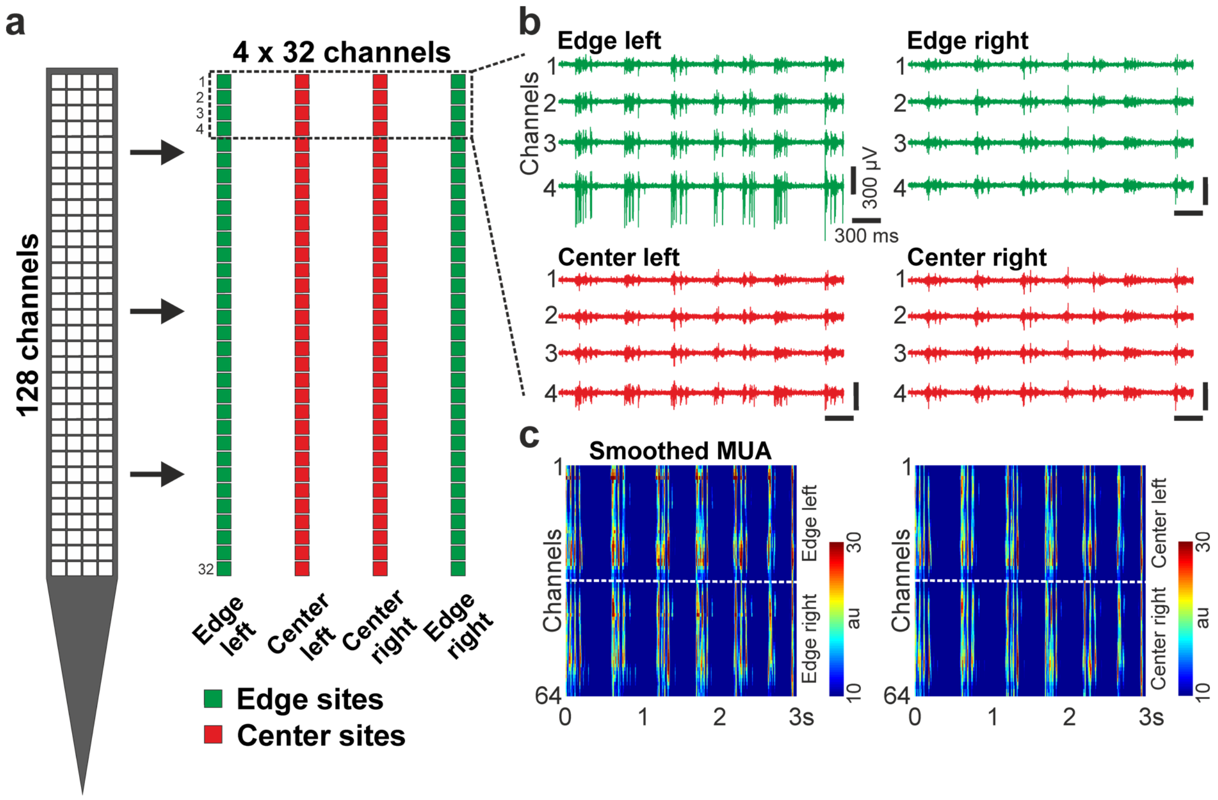

Supplementary Figure 2. Grouping of edge (green) and center (red) sites. (a) Recordings obtained with the silicon probe (e.g., the 128-channel NeuroSeeker probe shown here) were separated into groups of channels (4 × 32 channels here) based on the position of recording sites (edge or center). (b) Sample three-second-long multiunit activity (MUA; 500-5000 Hz) traces recorded on the channels indicated with a dashed rectangle on panel a. (c) Rectified and smoothed MUA (50 Hz lowpass filter) recorded on all edge (left) and center (right) channels. The dashed white lines separate channels located on the left and right side of the probe. au, arbitrary unit.

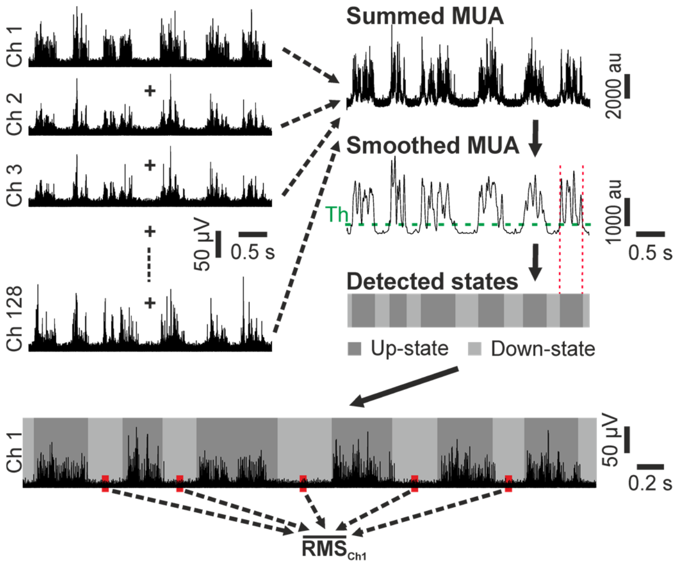

Supplementary Figure 3. The method used to estimate the noise level of silicon probe recordings in vivo. The onsets of up- and down-states were detected based on the MUA, then the root mean square (RMS) values of short segments of recordings were calculated in the center of down-states (low spiking activity) and averaged for each channel. A detailed description of the noise estimation method can be found in the Methods section.

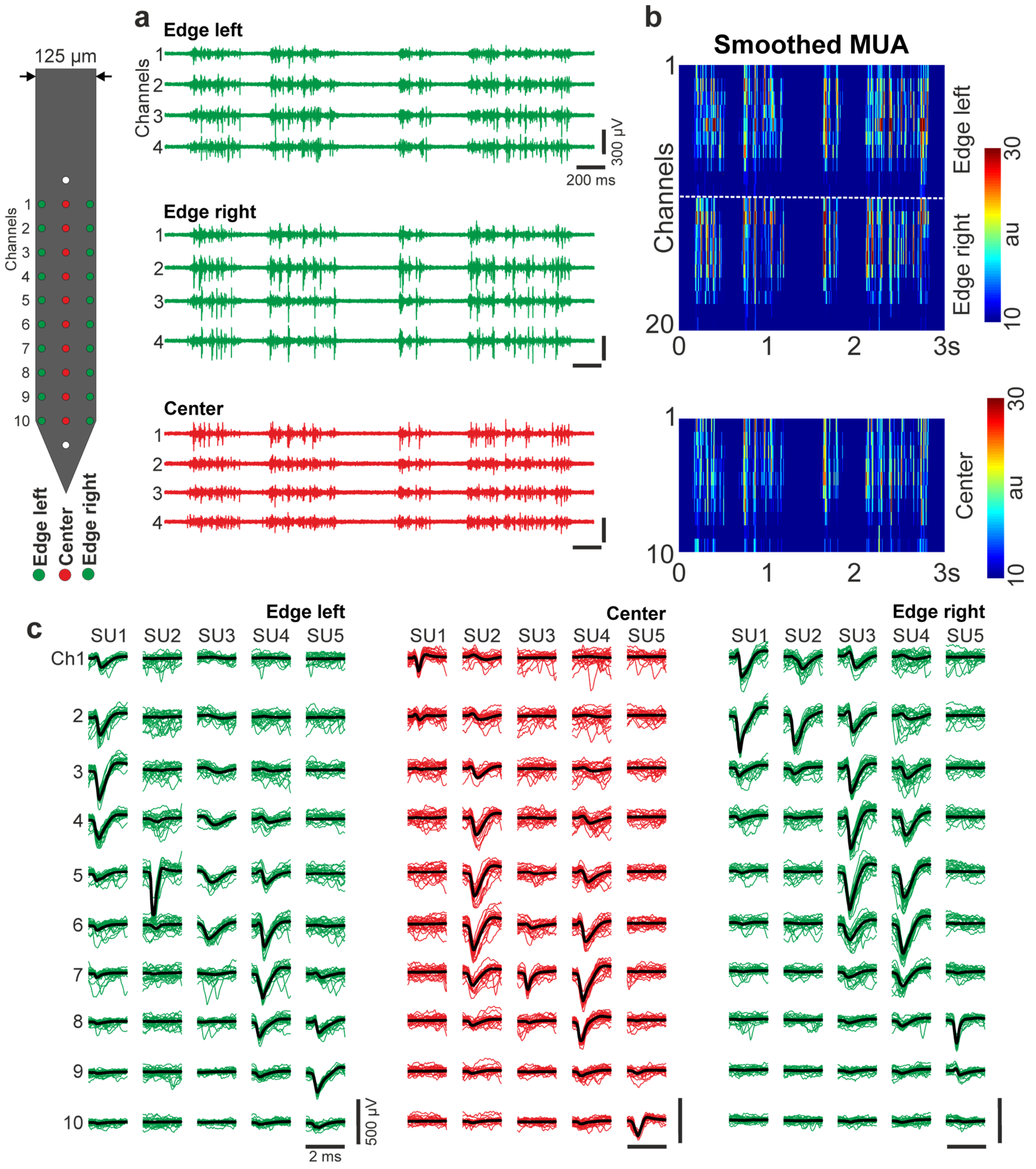

Supplementary Figure 4. Representative cortical data obtained with the 32-channel NeuroNexus probe from an anesthetized rat. (a) Examples of three-second-long multiunit activity (MUA; 500-5000 Hz) traces. Four channels for each site position are shown. (b) Rectified and smoothed MUA (50 Hz lowpass filter) recorded on all edge and center channels. The dashed white line separates channels located on the left and right edge of the probe (10 channels/site position; au, arbitrary unit). (c) Five exemplary single units (SU1-SU5) for each site position. For each single unit, twenty-five superposed individual wideband spikes (thin colored lines) and the average spike waveform (thick black line) are shown on ten channels.

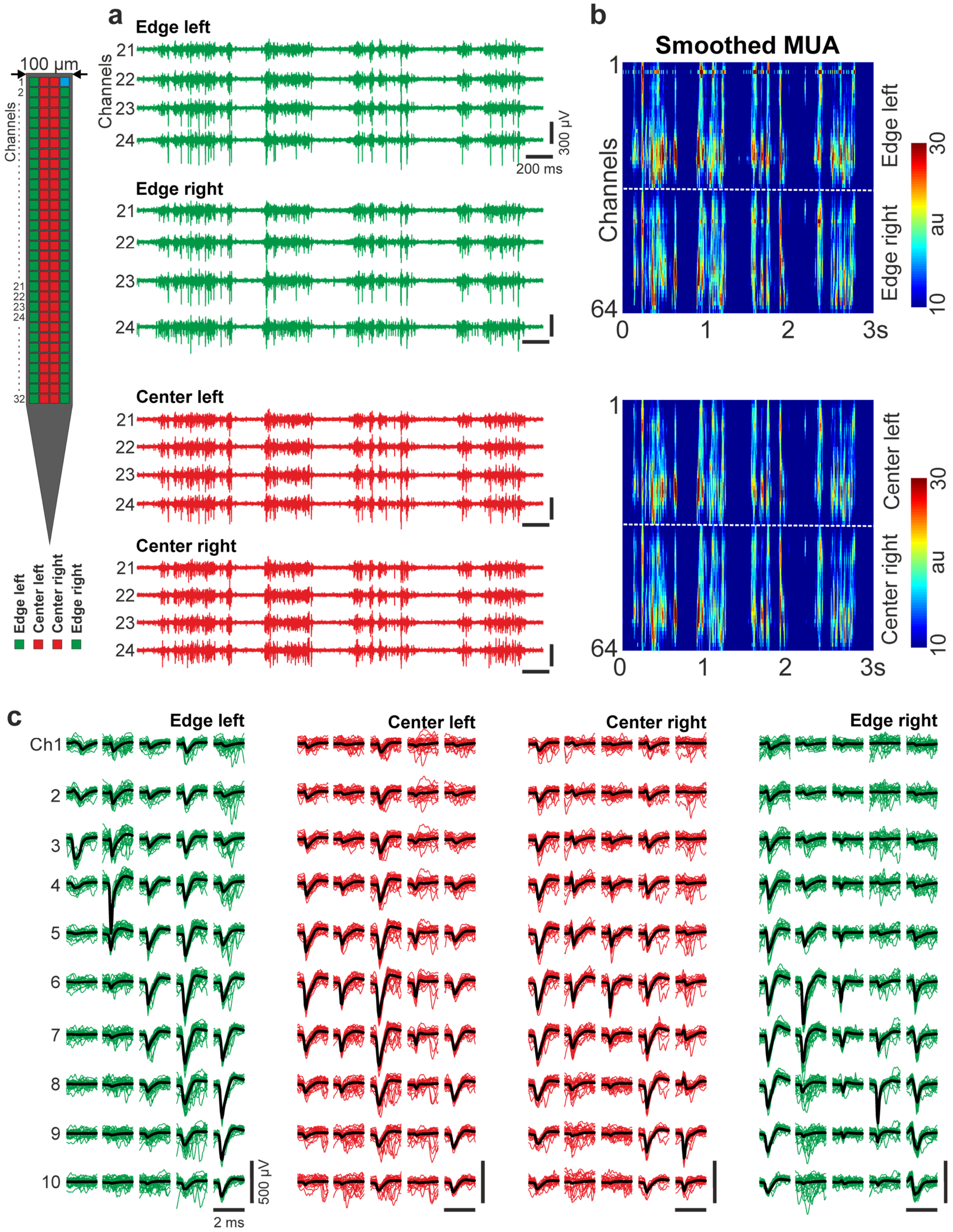

Supplementary Figure 5. Representative cortical data obtained with the 128-channel NeuroSeeker probe from an anesthetized rat. (a) Examples of three-second-long multiunit activity (MUA; 500-5000 Hz) traces. Four channels for each site position are shown. (b) Rectified and smoothed MUA (50 Hz lowpass filter) recorded on all edge and center channels. The dashed white lines separate channels located on the left and right side of the probe (32

channels/site position; au, arbitrary unit). (c) Five exemplary single units (columns) for each site position. For each single unit, twenty-five superposed individual wideband spikes (thin colored lines) and the average spike waveform (thick black line) are shown on ten adjacent channels.

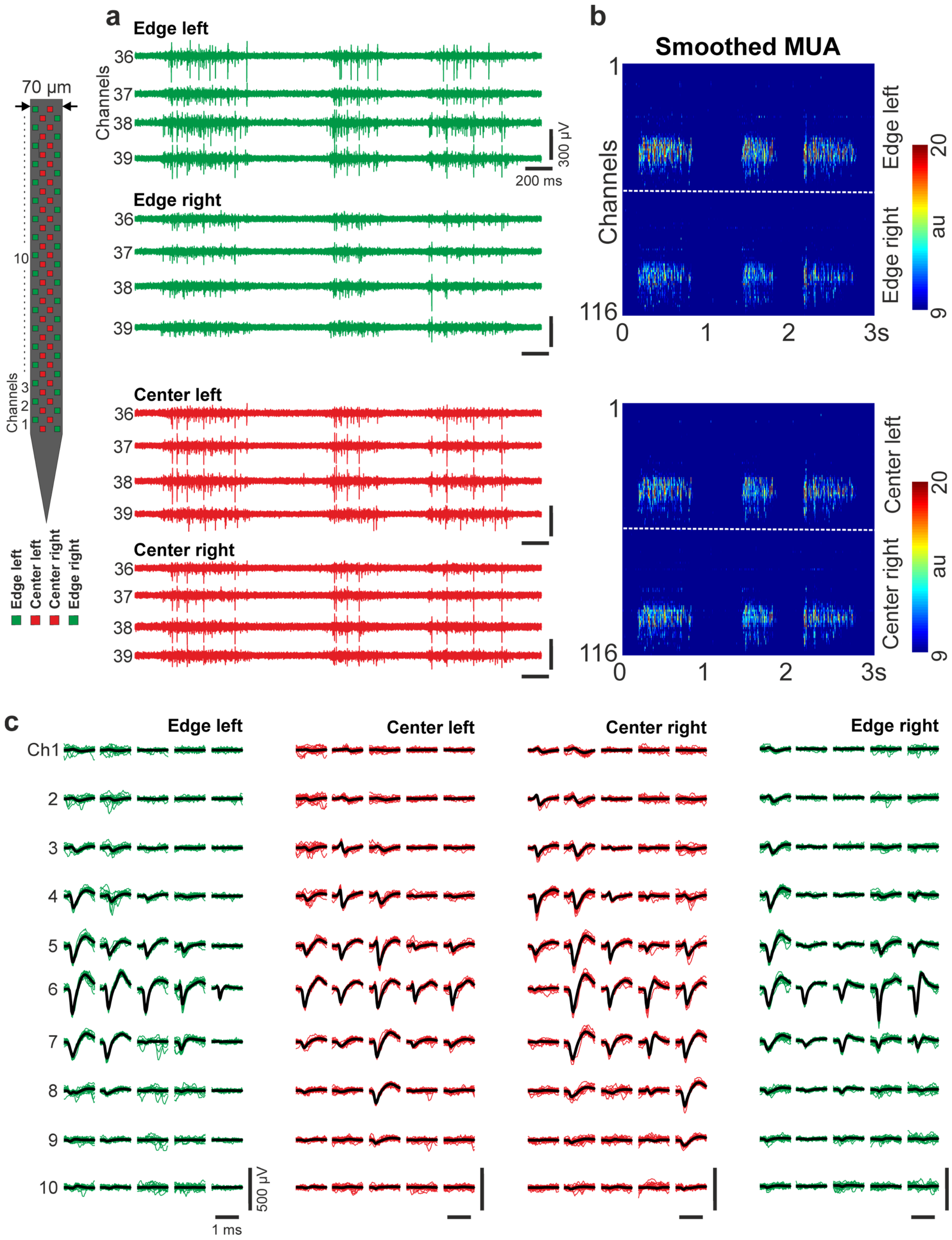

Supplementary Figure 6. Representative cortical data obtained with the 70-µm-wide Neuropixels probe from an anesthetized rat. (a) Examples of three-second-long multiunit activity (MUA; 500-5000 Hz) traces. Four channels for each site position are shown. (b) Rectified and smoothed MUA (50 Hz lowpass filter) recorded on all edge and center channels. The dashed white lines separate channels located on the left and right side of the probe (58 channels/site position; au, arbitrary unit). (c) Five exemplary single units(columns) for each site position. For each single unit, twenty-five superposed individual spikes (thin colored lines,

AP band) and the average spike waveform (thick black line) are shown on ten adjacent channels.

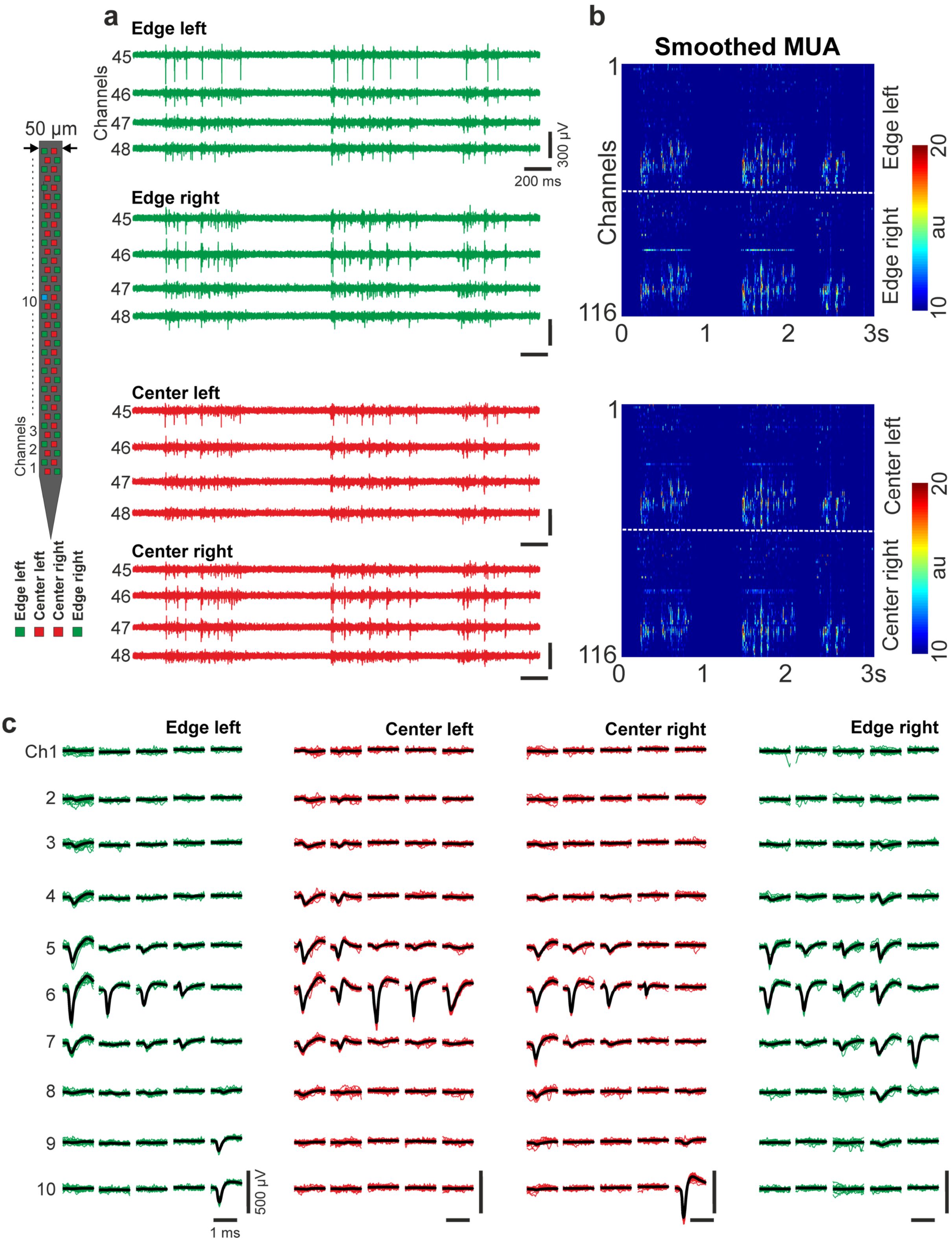

Supplementary Figure 7. Representative cortical data obtained with the 50-µm-wide Neuropixels probe from an anesthetized rat. (a) Examples of three-second-long multiunit activity (MUA; 500-5000 Hz) traces. Four channels for each site position are shown. (b) Rectified and smoothed MUA (50 Hz lowpass filter) recorded on all edge and center channels. The dashed white lines separate channels located on the left and right side of the probe (58

channels/site position; au, arbitrary unit). (c) Five exemplary single units (columns) for each site position. For each single unit, twenty-five superposed individual spikes (thin colored lines, AP band) and the average spike waveform (thick black line) are shown on ten adjacent channels.

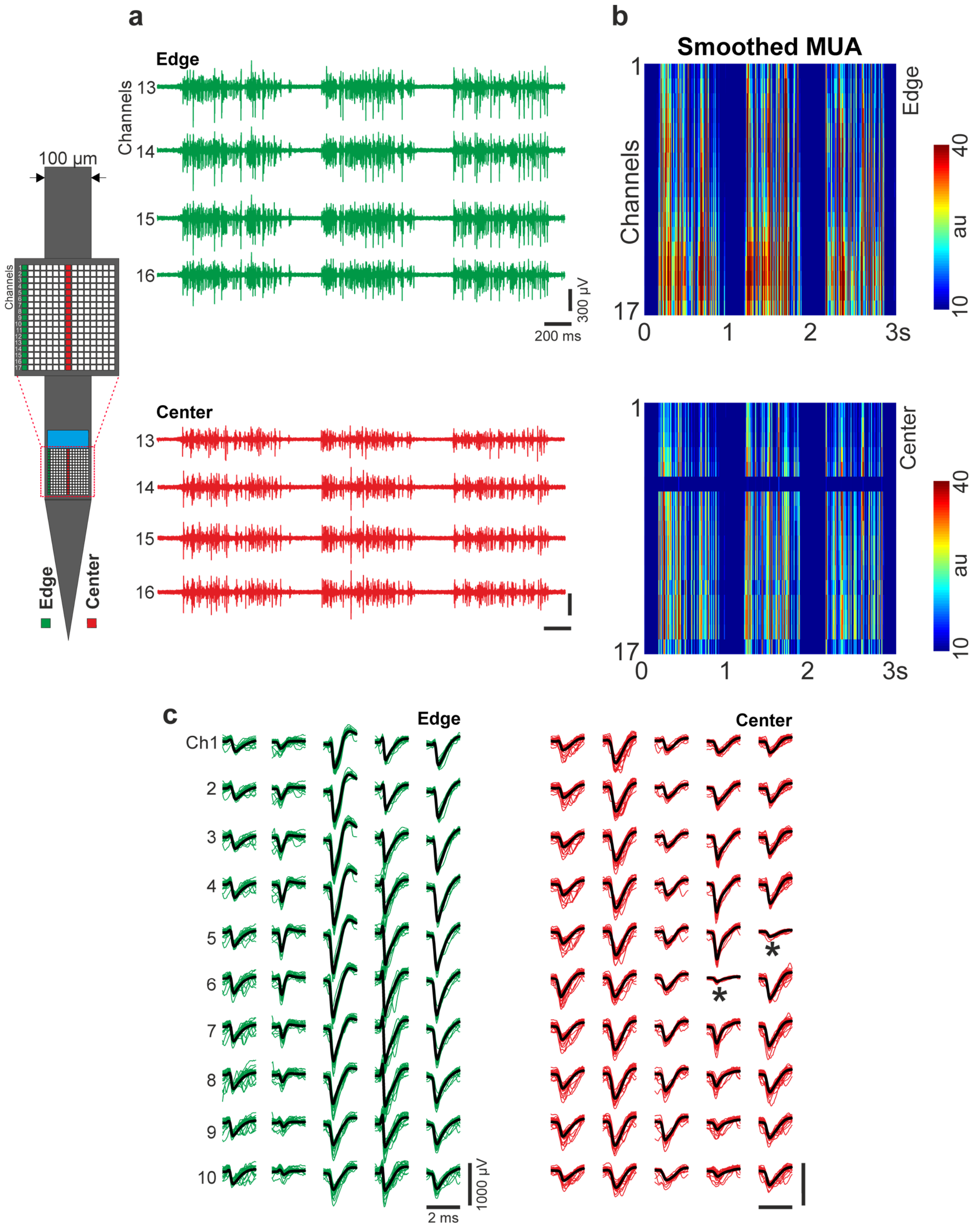

Supplementary Figure 8. Representative cortical data obtained with the 255-channel NeuroSeeker probe from an anesthetized rat. (a) Examples of three-second-long multiunit activity (MUA; 500-5000 Hz) traces. Four channels for each site position are shown. (b) Rectified and smoothed MUA (50 Hz lowpass filter) recorded on all left edge and center channels (17 channels/site position; au, arbitrary unit). (c) Five exemplary single units (columns) for each site position. For each single unit, twenty-five superposed individual wideband spikes (thin colored lines) and the average spike waveform (thick black line) are shown on ten adjacent channels. Asterisks indicate unfunctional channels.

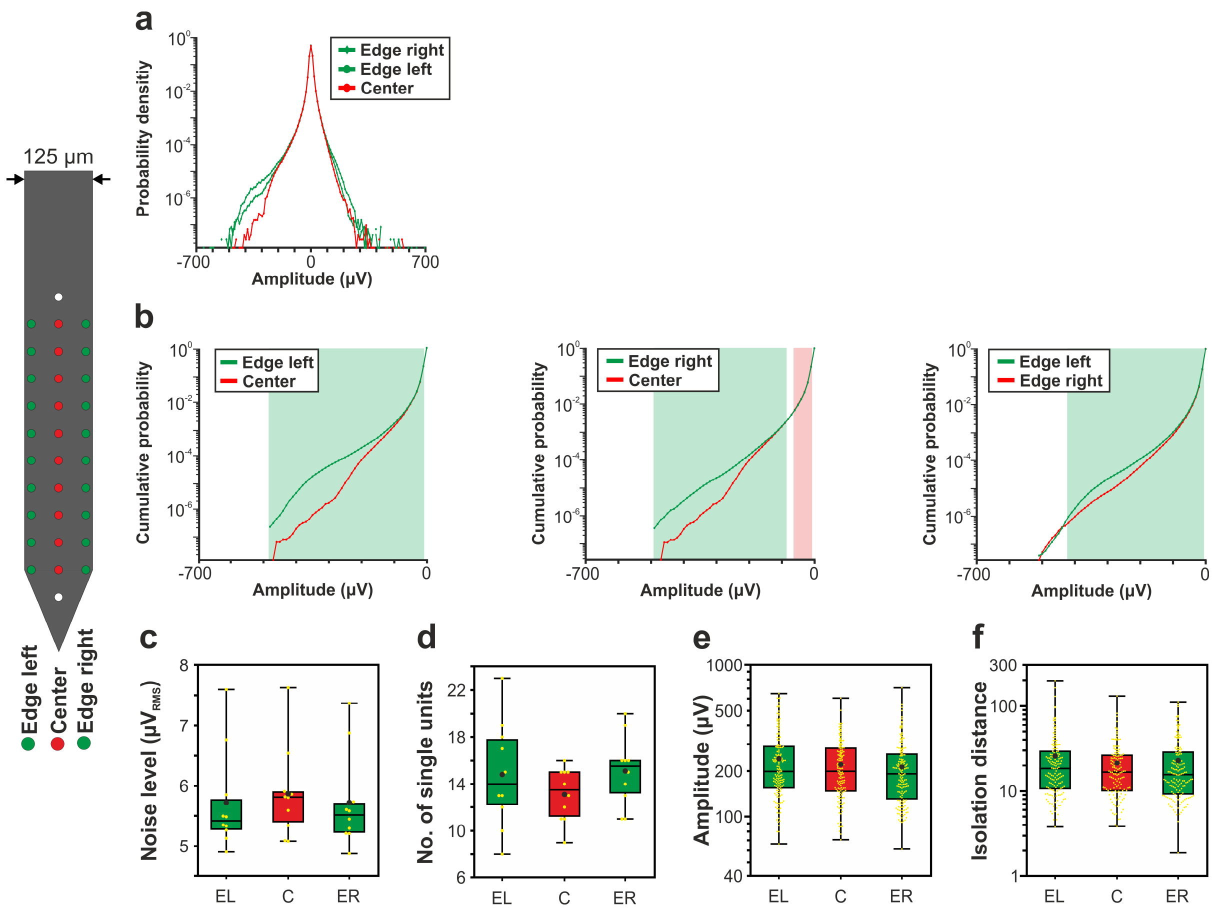

Supplementary Figure 9. *Comparison of the signal quality provided by left/right edge (green) and center (red) sites of the 32-channel NeuroNexus silicon probe.* (a) Probability density functions estimated from the amplitude of samples recorded in the 500 – 5000 Hz frequency range (n = 10 recordings). Probability values lower than 10^{-9} are not shown. (b) Pairs of cumulative amplitude distributions of all site positions calculated in the negative amplitude range. Green and red shaded areas indicate significant differences between distributions of site positions (e.g., green background shows that at the particular amplitudes there were significantly more samples in the cumulative distribution function represented by green color). (c) Estimated *in vivo* noise level. (d) Single unit yield (n = 430). (e) Peak-to-peak amplitude of the averaged single unit spike waveforms. (f) Isolation distance of the single unit clusters. All boxplots in the Supplementary material are presented as follows. The middle line indicates the median, while the boxes correspond to the 25th and 75th percentile. Whiskers mark the minimum and maximum values. The average is depicted with a black dot, while individual values are indicated with smaller yellow dots. Note that most data are plotted on a logarithmic scale.

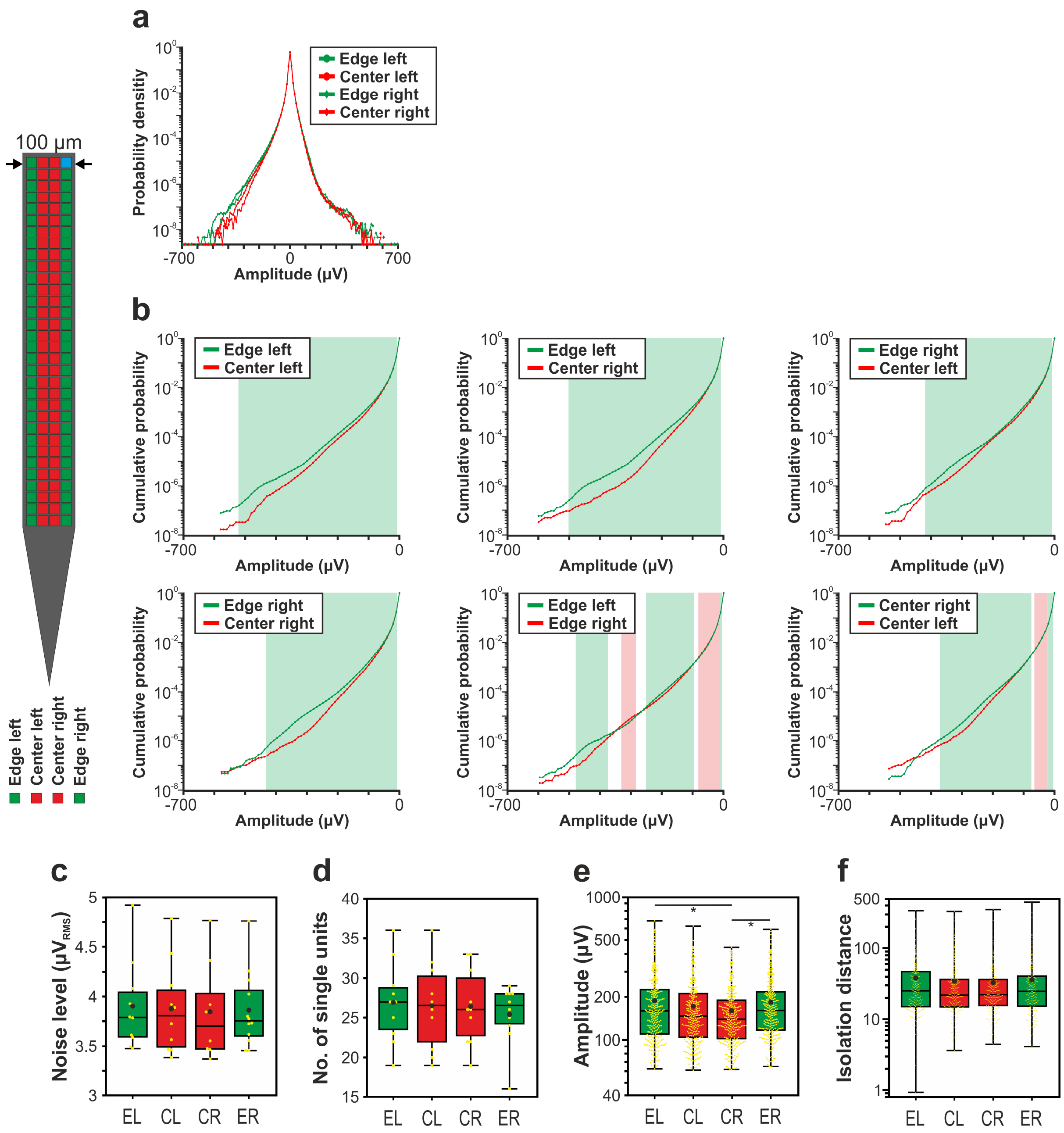

Supplementary Figure 10. *Comparison of the signal quality provided by left/right edge (green) and left/right center (red) sites of the 128-channel NeuroSeeker silicon probe.* (a) Probability density functions estimated from the amplitude of samples recorded in the 500 – 5000 Hz frequency range (n = 10 recordings). Probability values lower than 10^{-9} are not shown. (b) Pairs of cumulative amplitude distributions of all site positions calculated in the negative amplitude range. Green and red shaded areas indicate significant differences between distributions of site positions. (c) Estimated *in vivo* noise level. (d) Single unit yield (n = 1052). (e) Peak-to-peak amplitude of the averaged single unit spike waveforms. (f) Isolation distance of the single unit clusters. Note that most data are plotted on a logarithmic scale. * $p < 0.05$.

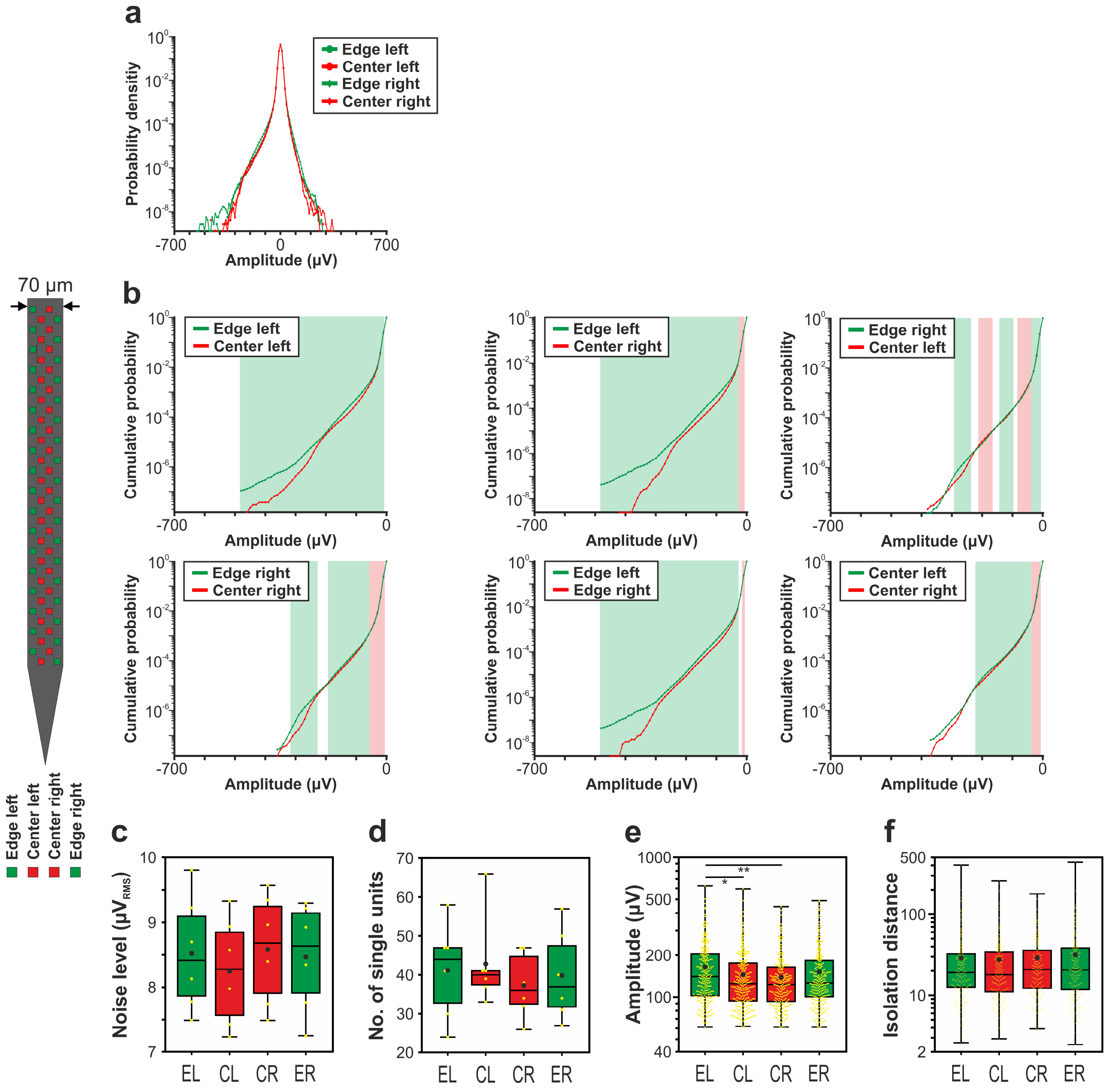

Supplementary Figure 11. *Comparison of the signal quality provided by left/right edge (green) and left/right center (red) sites of the 70-µm-wide Neuropixels probe.* (a) Probability density functions estimated from the amplitude of samples recorded in the 500 – 5000 Hz frequency range (n = 6 recordings). Probability values lower than 10^{-9} are not shown. (b) Pairs of cumulative amplitude distributions of all site positions calculated in the negative amplitude range. Green and red shaded areas indicate significant differences between distributions of site positions. (c) Estimated *in vivo* noise level. (d) Single unit yield (n = 967). (e) Peak-to-peak amplitude of the averaged single unit spike waveforms. (f) Isolation distance of the single unit clusters. Note that most data are plotted on a logarithmic scale. * $p < 0.05$; ** $p < 0.01$.

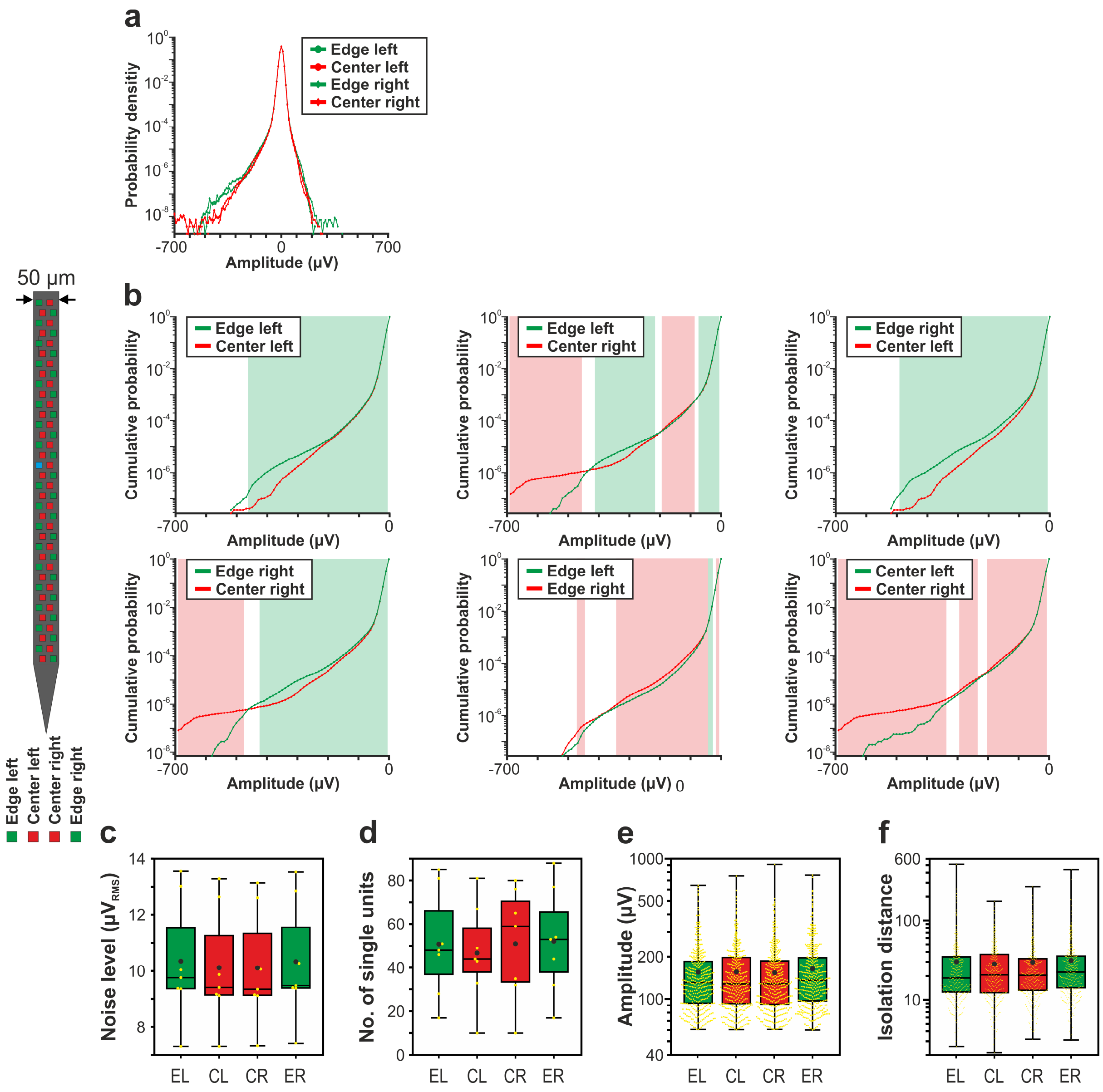

Supplementary Figure 12. *Comparison of the signal quality provided by left/right edge (green) and left/right center (red) sites of the 50-µm-wide Neuropixels probe.* (a) Probability density functions estimated from the amplitude of samples recorded in the 500 – 5000 Hz frequency range (n = 7 recordings). Probability values lower than 10^{-9} are not shown. (b) Pairs of cumulative amplitude distributions of all site positions calculated in the negative amplitude range. Green and red shaded areas indicate significant differences between distributions of site positions. (c) Estimated *in vivo* noise level. (d) Single unit yield (n = 1405). (e) Peak-to-peak amplitude of the averaged single unit spike waveforms. (f) Isolation distance of the single unit clusters. Note that most data are plotted on a logarithmic scale.

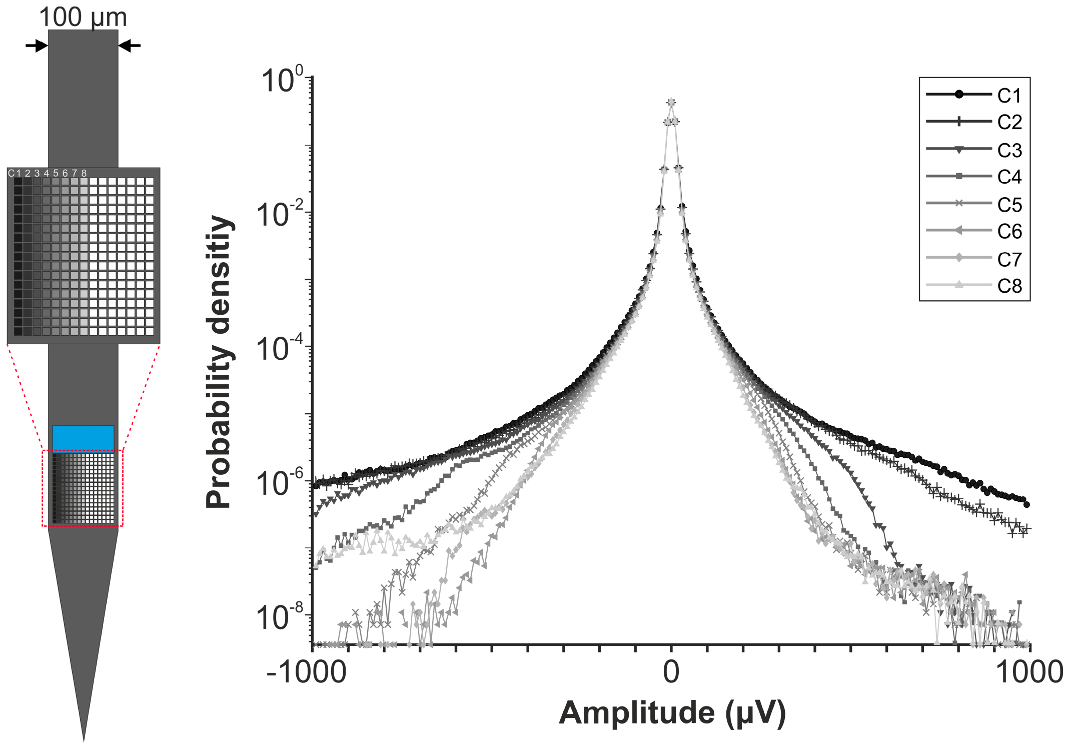

Supplementary Figure 13. *Comparison of the signal quality provided by eight columns of recordings sites of the 255-channel NeuroSeeker silicon probe.* Probability density functions estimated from the amplitude of samples recorded in the 500 – 5000 Hz frequency range (n = 21 recordings). Columns of recording sites on the left are color-coded (Column(C)1-C8: from dark gray to light gray; C1 corresponds to edge sites, while C8 represents the column of center sites). The probability density functions on the right are plotted with the same color scheme. Note that data are plotted on a logarithmic scale.

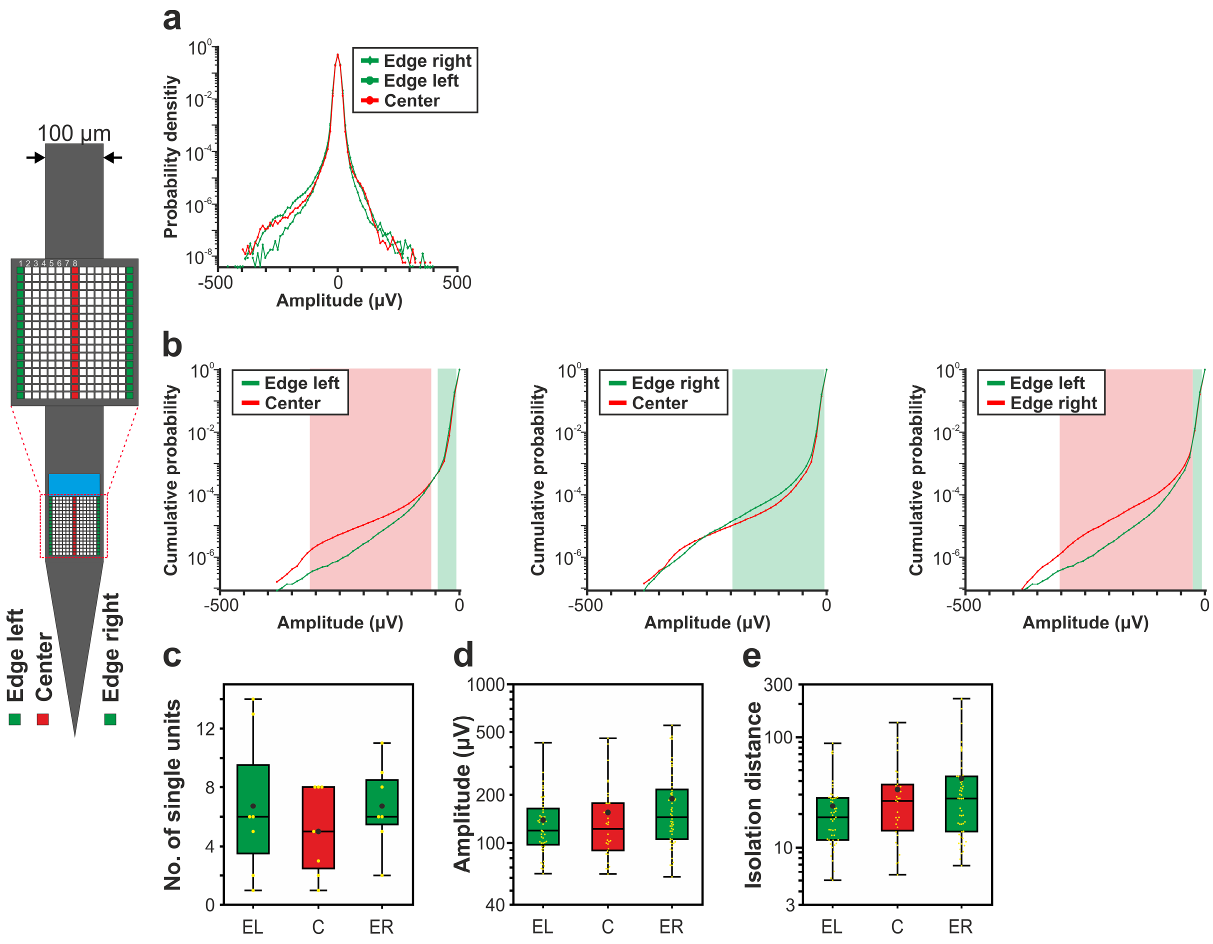

Supplementary Figure 14. *Comparison of the signal quality provided by left/right edge (green) and center (red) sites of the online available 255-channel silicon probe data (www.kampff-lab.org/ultra-dense-survey).* (a) Probability density functions estimated from the amplitude of samples recorded in the 500 – 5000 Hz frequency range (n = 7 recordings). (b) Pairs of cumulative amplitude distributions of all site positions calculated in the negative range. Green and red shaded areas indicate significant differences between distributions of site positions. (c) Single unit yield (n = 129). (d) Peak-to-peak amplitude of the averaged single unit spike waveforms. (e) Isolation distance of the single unit clusters. Note that most data are plotted on a logarithmic scale.

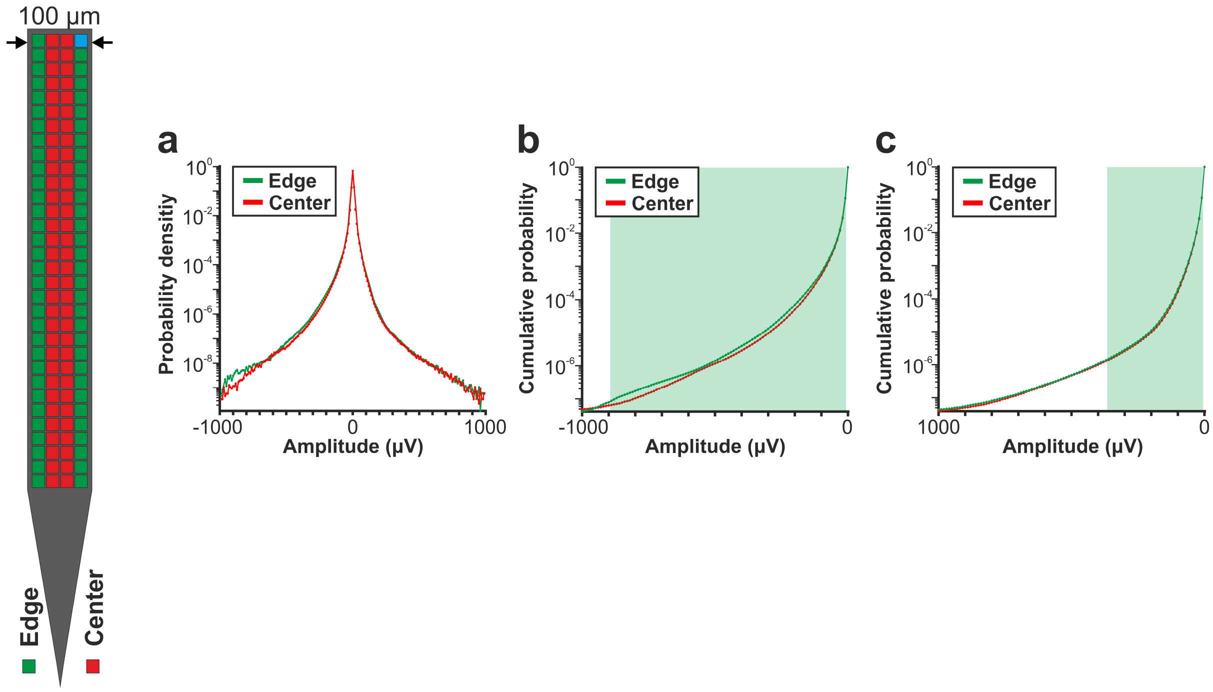

Supplementary Figure 15. *Comparison of the signal quality provided by edge (green) and center (red) sites using a larger dataset obtained with the 128-channel NeuroSeeker silicon probe.* (a) Probability density functions estimated from the amplitude of samples recorded in the 500 – 5000 Hz frequency range (n = 179 recordings). (b) Cumulative amplitude distributions calculated in the negative range. (c) Reverse cumulative distributions calculated in the positive amplitude range. Green shaded areas in (b) and (c) indicate amplitudes with significantly higher numbers of edge samples.

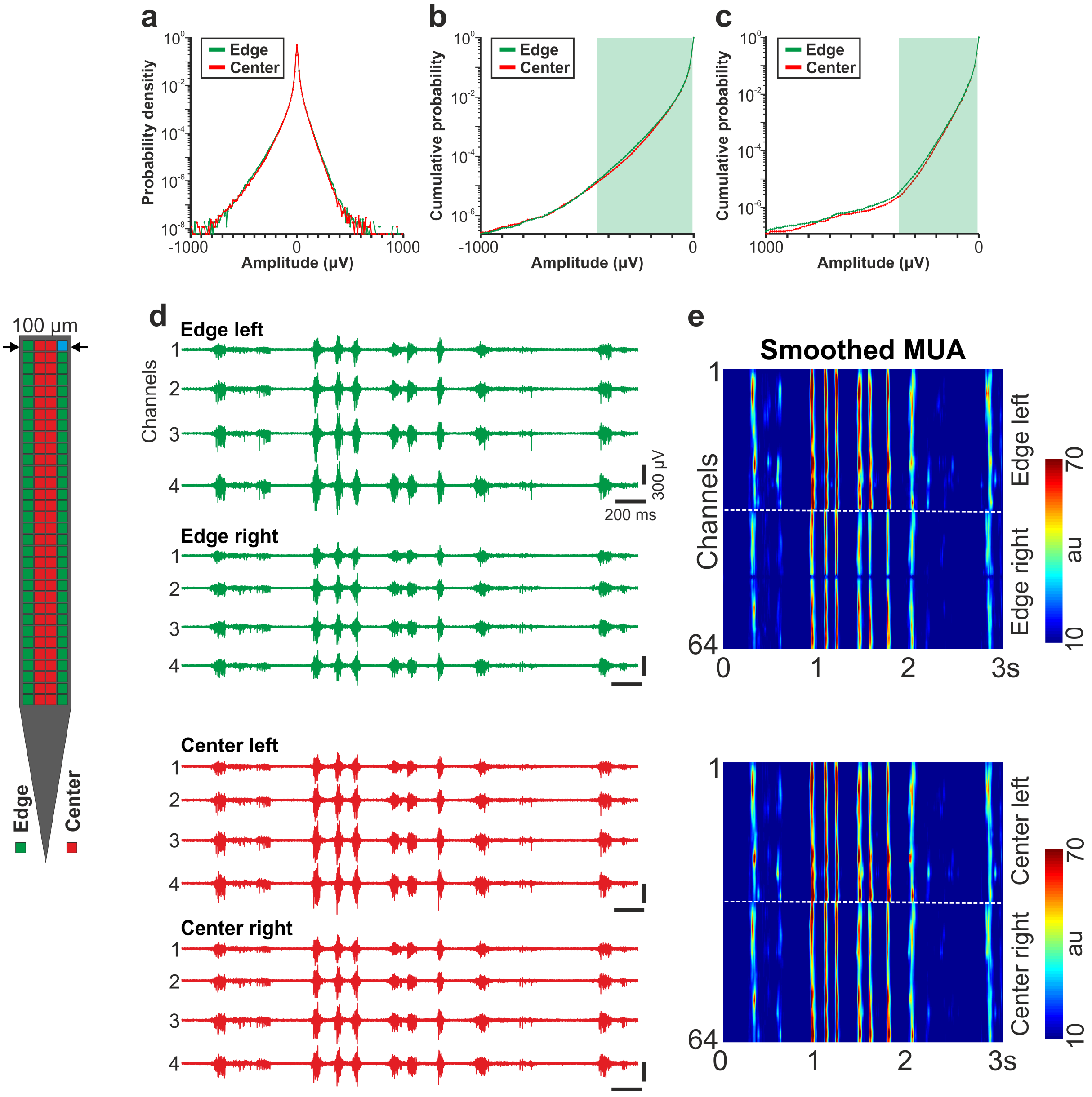

Supplementary Figure 16. *Comparison of the thalamic signal quality provided by left/right edge (green) and left/right center (red) sites of the 128-channel NeuroSeeker silicon probe.* (a) Probability density functions estimated from the amplitude of samples recorded in the 500 – 5000 Hz frequency range (n = 9 recordings). (b) Cumulative amplitude distributions calculated in the negative range. (c) Reverse cumulative distributions calculated in the positive amplitude range. Green shaded areas in (b) and (c) indicate amplitudes with significantly higher numbers of edge samples. Note that data are plotted on a logarithmic scale. (d) Examples of three-second-long thalamic multiunit activity (MUA; 500-5000 Hz) traces. Four channels for each site position are shown. (e) Rectified and smoothed MUA (50 Hz lowpass filter) recorded on all edge and center channels. The dashed white lines separate channels located on the left and right side of the probe (32 channels/site position; au, arbitrary unit). * $p < 0.05$; *** $p < 0.001$.

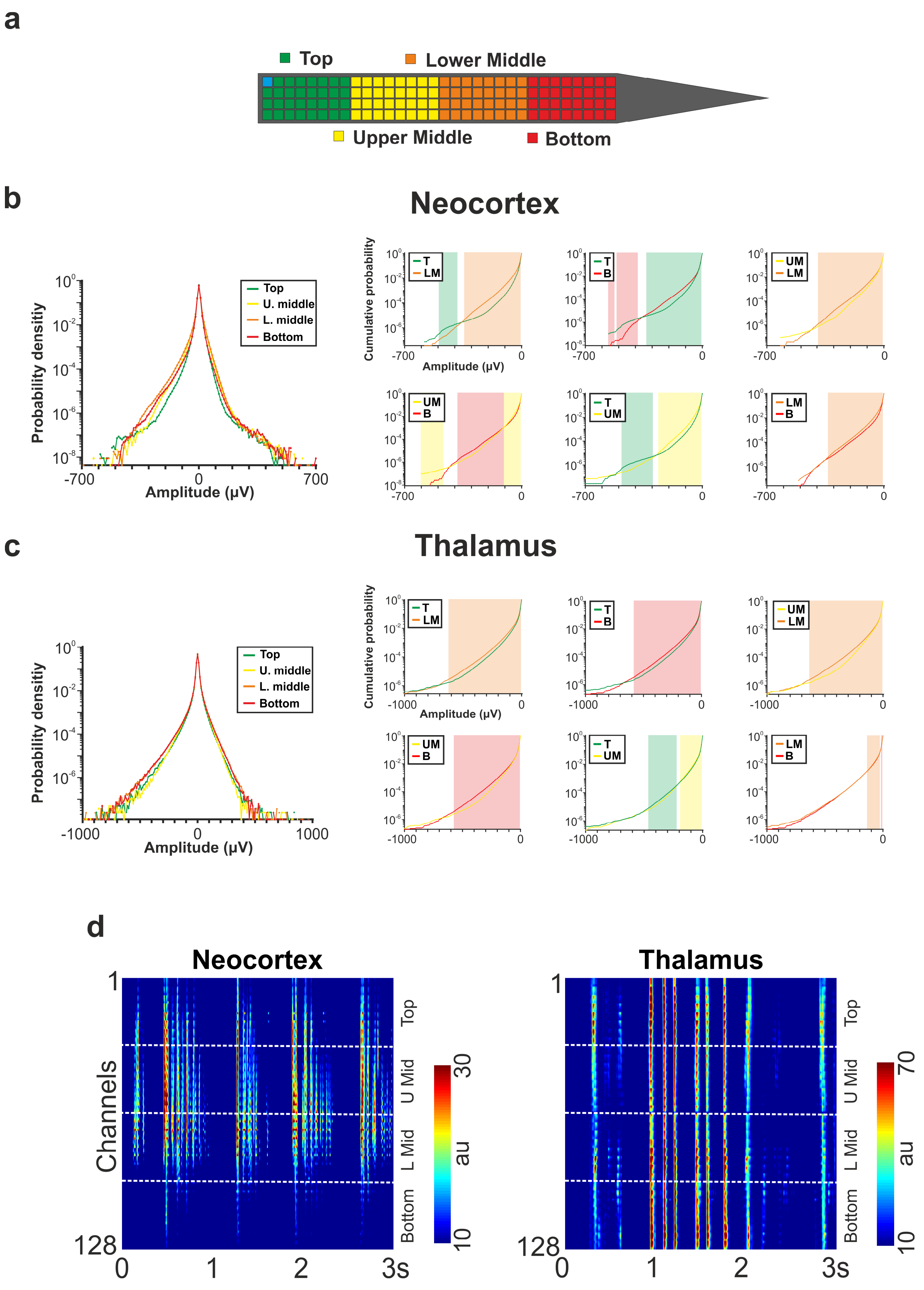

Supplementary Figure 17. *Results of the analysis based on longitudinal site positions.* (a) The recording sites of the 128-channel probe were grouped according to their longitudinal position

(32-channel/site position). Four site groups were constructed with the bottom sites located closest to the probe tip. (b-c) Probability density functions (left) estimated from the amplitude of samples recorded in the 500 – 5000 Hz frequency range, and pairs of cumulative amplitude distributions (right) of all site positions calculated in the negative range for (b) cortical data (n = 10 recordings) and for the (c) thalamic data (n = 9 recordings), separated by longitudinal site position. Note that data are plotted on a logarithmic scale. Colored shaded areas in (b) and (c) indicate significant differences between distributions of site positions (the same color scheme was used as for the site positions). (d) Three-second-long rectified and smoothed (50 Hz lowpass filter) multiunit activity recorded in the cortex (left) and in the thalamus (right). Note that, compared to the thalamus, cortical spiking activity is usually not recorded simultaneously on all sites. T – top, UM – upper middle, LM – lower middle, B – bottom.

Probe type	No. of recording sites	No. of sites in separated recordings	No. of analysed recordings	No. of rats	No. of penetrations	Average recording length (min)	Reference
255-channel NeuroSeeker probe	255	17	7	3	3	27	Dimitriadis et al., 2018, bioRxiv 275818

Supplementary Table 1. Details of the experiments and recordings of the 255-channel silicon probe data available online (www.kampff-lab.org/ultra-dense-survey). Cortical recordings with the following identifiers were used in the analysis: Co1, Co2, Co3, Co5, CoP1, CoP2, CoP3.

Probe type	No. of recording sites	No. of sites in separated recordings	No. of analysed recordings	No. of rats	No. of penetrations	Type of anesthesia	Average recording length (min)
128-channel NeuroSeeker probe	128	32	9	3	3	Ketamine/xylazine	12

Supplementary Table 2. Details of the thalamic experiments and recordings with the 128-channel probe.

	32-channel NN probe		
	Edge Left	Center	Edge Right
RMS of amplitudes	**12.58**	11.96	11.94
No. of single units	14.80 ± 3.73	13.10 ± 2.28	**15.10 ± 2.99**
Amplitude of single units (µV)	**240.46 ± 127.62**	220.11 ± 97.39	211.90 ± 105.30
Isolation distance	**25.84 ± 26.90**	21.35 ± 17.3	22.94 ± 20.43
Noise level (μV_{RMS})	5.72 ± 0.83	**5.87 ± 0.75**	5.72 ± 0.79

Supplementary Table 3. Mean ± standard deviation of the calculated features for the 32-channel NeuroNexus probe. The root mean square (RMS) of amplitudes was calculated by pooling the samples of all recordings. For each feature, the largest value is indicated in bold.

	128-channel NS probe			
	Edge Left	Center Left	Center Right	Edge Right
RMS of amplitudes	11.68	11.23	11.16	**11.70**
No. of single units	**26.90 ± 5.07**	26.50 ± 5.52	26.40 ± 4.77	25.40 ± 3.84
Amplitude of single units (μV)	**186.90 ± 108.42**	170.63 ± 92.56	158.65 ± 77.20	182.85 ± 95.12
Isolation distance	**38.06 ± 40.68**	34.41 ± 39.16	32.63 ± 38.00	36.04 ± 43.68
Noise level (μV_{RMS})	**3.90 ± 0.45**	3.87 ± 0.46	3.85 ± 0.44	3.86 ± 0.42

Supplementary Table 4. Mean ± standard deviation of the calculated features for the 128-channel NeuroSeeker probe. The root mean square (RMS) of amplitudes was calculated by pooling the samples of all recordings.

	70 µm NP probe			
	Edge Left	Center Left	Center Right	Edge Right
RMS of amplitudes	**9.79**	9.43	9.63	9.54
No. of single units	41.17 ± 12.42	**42.83 ± 11.74**	37.33 ± 8.43	39.83 ± 11.62
Amplitude of single units (µV)	**163.57 ± 85.77**	143.77 ± 74.13	137.68 ± 65.27	150.58 ± 79.36
Isolation distance	28.92 ± 36.04	27.60 ± 32.32	28.98 ± 26.79	**31.63 ± 38.03**
Noise level (μV_{RMS})	8.52 ± 0.89	8.24 ± 0.84	**8.58 ± 0.85**	8.46 ± 0.83

Supplementary Table 5. Mean ± standard deviation of the calculated features for the 70-µm-wide Neuropixels probe. The root mean square (RMS) of amplitudes was calculated by pooling the samples of all recordings.

	50 µm NP probe			
	Edge Left	Center Left	Center Right	Edge Right
RMS of amplitudes	10.95	10.82	10.89	**11.02**
No. of single units	50.86 ± 25.08	46.71 ± 22.91	51.00 ± 25.9	**52.14 ± 24.55**
Amplitude of single units (µV)	155.24 ± 91.12	155.96 ± 92.13	154.06 ± 93.45	**164.06 ± 100.24**
Isolation distance	30.12 ± 39.29	28.30 ± 24.26	29.72 ± 32.68	**31.28 ± 36.19**
Noise level (μV_{RMS})	**10.34 ± 2.20**	10.11 ± 2.11	10.10 ± 2.07	10.33 ± 2.14

Supplementary Table 6. Mean ± standard deviation of the calculated features for the 50-µm-wide Neuropixels probe. The root mean square (RMS) of amplitudes was calculated by pooling the samples of all recordings.

Probe type	No. of excluded single units	Total number of single units included	% of units excluded
32-channel NeuroNexus probe	6	430	1.38
128-channel NeuroSeeker probe	156	1052	12.91
Neuropixels probe (70 µm wide)	61	967	5.93
Neuropixels probe (50 µm wide)	95	1405	6.33
255-channel NeuroSeeker probe	27	599	4.31

Supplementary Table 7. Number and ratio of single units excluded from the analysis due to poor cluster quality.

Probe type	Original recordings	Separated recordings	Difference
32-channel NeuroNexus probe	212	430	2.03
128-channel NeuroSeeker probe	414	1052	2.54
Neuropixels probe (70 µm wide)	511	967	1.89
Neuropixels probe (50 µm wide)	713	1405	1.97

Supplementary Table 8. Total single unit yield obtained in the case of original recordings (all channels included) and in the case of separated recordings (with reduced channel counts). Single unit yields of separated recordings have been added together to estimate the redundancy among the sorted single units. The 255-channel probe has a significantly different layout; therefore the 255-channel data was excluded from this analysis.

	Edge/Center	Left Edge/Left Center/Right Center/Right Edge
RMS of amplitudes	Brown-Forsythe test of equal variance	-
Cumulative distribution	Binomial test	Binomial test
No. of single units	Mann-Whitney U test	Kruskal-Wallis test with post-hoc Dunn’s test with Bonferroni correction
Amplitude of single units (μV)	Mann-Whitney U test	Kruskal-Wallis test with post-hoc Dunn’s test with Bonferroni correction
Isolation distance	Mann-Whitney U test	Kruskal-Wallis test with post-hoc Dunn’s test with Bonferroni correction
Noise level (μV_{RMS})	Mann-Whitney U test	Kruskal-Wallis test with post-hoc Dunn’s test with Bonferroni correction

Supplementary Table 9. List of the statistical tests used in the study.

	255-channel NS probe			
	Column 1 (Edge)	Column 2	Column 3	Column 4
RMS of amplitudes	**23.67**	20.90	17.74	16.38

	255-channel NS probe			
	Column 5	Column 6	Column 7	Column 8 (Center)
RMS of amplitudes	15.44	14.77	14.35	14.08

Supplementary Table 10. Root mean square (RMS) of amplitudes for the first eight columns of recording sites of the 255-channel NeuroSeeker probe. See Supplementary Figure 13 for more details.

	255-channel NS probe		
	Edge Left	Center	Edge Right
RMS of amplitudes	**8.06**	7.35	7.82
No. of single units	**6.71 ± 5.02**	5.00 ± 3.06	**6.71 ± 2.93**
Amplitude of single units (μV)	138.48 ± 66.29	153.98 ± 99.49	**188.33 ± 118.54**
Isolation distance	23.65 ± 18.52	33.35 ± 30.16	**42.30 ± 48.72**

Supplementary Table 11. Mean ± standard deviation of the calculated features for the 255-channel silicon probe data available online (www.kampff-lab.org/ultra-dense-survey). The root mean square (RMS) of amplitudes was calculated by pooling the samples of all recordings.

	128-channel NS probe	
	Edge	Center
RMS of amplitudes	**8.72**	8.42

Supplementary Table 12. Root mean square (RMS) of amplitudes for the larger dataset (n = 179 recordings) obtained with the 128-channel NeuroSeeker probe. The RMS was calculated by pooling the samples of all recordings.

	128-channel NS probe			
	Edge Left	Center Left	Center Right	Edge Right
RMS of amplitudes	**8.74**	8.44	8.41	8.70

Supplementary Table 13. Root mean square (RMS) of amplitudes for the large dataset (n = 179 recordings) obtained with the 128-channel NeuroSeeker probe, separated by left and right side.

	128-channel NS probe	
	Edge	Center
RMS of amplitudes	**17.63**	17.17

Supplementary Table 14. Root mean square (RMS) of amplitudes for the thalamic dataset (n = 9 recordings) obtained with the 128-channel NeuroSeeker probe. The RMS was calculated by pooling the samples of all recordings.

	128-channel NS probe			
	Edge Left	Center Left	Center Right	Edge Right
RMS of amplitudes	**18.33**	17.49	16.84	16.86

Supplementary Table 15. Root mean square (RMS) of amplitudes for the thalamic dataset (n = 9 recordings) obtained with the 128-channel NeuroSeeker probe, separated by left and right side.

	Neocortex			
	Top	Upper Middle	Lower Middle	Bottom
RMS of amplitudes	9.40	12.19	**13.35**	10.35

Supplementary Table 16. Root mean square (RMS) of amplitudes for the neocortical data obtained with the 128-channel probe, separated by longitudinal site position. The RMS was calculated by pooling the amplitude of all recordings.

	Thalamus			
	Top	Upper Middle	Lower Middle	Bottom
RMS of amplitudes	15.85	16.89	**18.47**	18.24

Supplementary Table 17. Root mean square (RMS) of amplitudes for the thalamic data obtained with the 128-channel probe, separated by longitudinal site position. The RMS was calculated by pooling the amplitude of all recordings.
